# Improving hospital substance use care from the ground up and from the outside in

**DOI:** 10.17269/s41997-026-01154-5

**Published:** 2026-03-20

**Authors:** Maya Lowe, Landon “Morty” Morton, Eva Burrill, J. Mariah Hughes, Emily MacAdam, William Nevers, Jo Parker, Caitlin Sampson, Thomas D. Brothers

**Affiliations:** 1 Faculty of Medicine, Dalhousie University, Halifax, NS Canada; 2 Mainline Needle Exchange, Halifax, NS Canada; 3 Social Work, Nova Scotia Health, Halifax, NS Canada; 4 Division of Infectious Diseases, Department of Medicine, Dalhousie University, Halifax, NS Canada; 5 Addiction Medicine Consult Service, Nova Scotia Health, Halifax, NS Canada; 6 Infectious Diseases, Health PEI, Charlottetown, PEI Canada; 7 Nursing, Nova Scotia Health, Halifax, NS Canada; 8 Pharmacy, Nova Scotia Health, Halifax, NS Canada; 9 Division of General Internal Medicine, Department of Medicine, Dalhousie University and Nova Scotia Health, Halifax, NS Canada

**Keywords:** Hospital, Acute care, Substance use, Addiction, Harm reduction, Harm reduction, Hôpitaux, Soins de courte durée, Utilisation de substances, Dépendance, Réduction des méfaits

## Abstract

**Setting:**

The Queen Elizabeth II (QEII) Health Sciences Centre in Halifax, Nova Scotia, is an academic, tertiary care hospital that lacked policies related to unregulated substance use, harm reduction, and addiction care. Substance use care was of poor quality and inconsistent, and the community-based standard-of-care was unavailable to hospitalized patients.

**Intervention:**

A group of frontline hospital- and community-based healthcare providers, trainees, harm reduction workers, and people with lived experience organized to navigate this “policy vacuum”. We invited community-based groups and resources into the hospital, developed informal policies and procedures to standardize care, and trained ourselves and others in substance use care best practices.

**Outcomes:**

We introduced several harm reduction initiatives from the community—including take-home naloxone kits, needle and syringe distribution, and oral and injectable opioid agonist treatment—without institutional policy support. We drafted our own informal harm reduction policies for the internal medicine inpatient unit, holding a focus group with people with lived experience for feedback and revision. This work contributed to funding for an addiction medicine consultation service and an institutional commitment to implement harm reduction-oriented substance use policies for healthcare settings across the province.

**Implications:**

Despite a lack of institutional policies or buy-in from senior leadership, harm reduction measures can be implemented in hospitals from the bottom-up and the outside-in—by healthcare providers organizing, leveraging existing community resources, and listening to people who use drugs. Clinicians at other hospitals could model our collaborative approach to improve care and push their health systems towards institutional change.

**Supplementary Information:**

The online version contains supplementary material available at https://doi.org/10.17269/s41997-026-01154-5.

## Résumé

**Lieu:**

Le Queen Elizabeth II Health Sciences Centre de Halifax (NouvelleÉcosse) est un hôpital universitaire de soins tertiaires qui n’avait pas de politiques liées à l’usage de substances non réglementées, à la réduction des méfaits ou aux soins des dépendances. Les soins en usage de substances y étaient de mauvaise qualité et manquaient de cohérence, et les patientes et patients hospitalisés n’avaient pas accès aux normes de soins axés sur la communauté.

**Intervention:**

Nous sommes un groupe de professionnelles et de professionnels de santé de première ligne en milieu hospitalier et communautaire, de stagiaires, d’intervenantes et d’intervenants en réduction des méfaits et de personnes ayant une expérience vécue qui nous sommes organisés pour combler ce vide. Nous avons invité à l’hôpital des groupes et des ressources de proximité, élaboré des politiques et procédures informelles de normalisation des soins, et reçu et donné de la formation sur les pratiques exemplaires en matière de soins en usage de substances.

**Résultats:**

Nous avons introduit plusieurs initiatives de réduction des méfaits venant de la communauté, dont des trousses maison de naloxone, la distribution d’aiguilles et de seringues et le traitement par agonistes opioïdes par voie orale et injectable, sans le soutien de l’établissement à ces réformes. Nous avons élaboré nos propres politiques informelles de réduction des méfaits pour l’unité d’hospitalisation en médecine interne en organisant un groupe de discussion avec des personnes ayant une expérience vécue pour obtenir leurs réactions et corriger le tir au besoin. Ce travail a contribué au financement d’un service de consultation en médecine des dépendances et mené à l’engagement de l’hôpital à mettre en oeuvre des politiques d’usage de substances axées sur la réduction des méfaits pour les milieux de soins de toute la province.

**Implications:**

Même en l’absence de politiques institutionnelles et sans l’aval de la haute direction, des mesures de réduction des méfaits dans les hôpitaux peuvent être mises en oeuvre du bas vers le haut et de l’extérieur vers l’intérieur lorsque les professionnelles et les professionnels de santé s’organisent, exploitent les ressources communautaires existantes et écoutent les personnes faisant usage de drogue. Le personnel clinique d’autres hôpitaux peut s’inspirer de notre démarche concertée pour améliorer les soins et opérer un changement institutionnel dans ses systèmes de santé.

## Introduction

Hospitals can be dangerous environments for people who use drugs. Systemic issues like stigma, abstinence-based policies, lack of harm reduction supports, and clinicians’ knowledge gaps contribute to inadequate pain and withdrawal management, and negative care experiences. Many people who use drugs delay presentation for care even when very sick and treat the emergency department as a “last resort” (Brothers et al., 2023a; Harris et al., 2020; Heller et al., 2004). Hospital inpatients must hide their drug use and use in more dangerous ways (e.g. in locked bathrooms) or leave hospital prematurely, before medically advised (Brothers et al., 2022; Lewer et al., 2023; Nolan et al., 2022). This hostile environment and poor-quality care lead to avoidable death, disability, and suffering for people who use drugs (Brothers et al., 2021b; Lennox et al., 2021; McNeil et al., 2014).

The Queen Elizabeth II (QEII) Health Sciences Centre in Halifax is the largest hospital in Nova Scotia and is the province’s academic and tertiary referral centre. A 2015–2017 needs assessment at the QEII found that among patients with injection drug use-associated endocarditis, at least two in three patients had concerns about undertreated pain or withdrawal documented in their chart and one in three patients was known to be using drugs while admitted to hospital (Brothers et al., 2022). Fewer than half of patients with untreated opioid use disorder were offered opioid agonist treatment (e.g. methadone, buprenorphine). Additionally, one in four patients had sterile injecting equipment seized from them, and no patients were provided with sterile injecting equipment in return (Brothers et al., 2022).

In 2015, nine distinct regional health authorities were amalgamated into a singular Nova Scotia Health Authority (NSHA). This change created a “policy vacuum” where any existing policies pertaining to people who use drugs in hospitals were no longer recognized, and no new policies were developed (Hyshka et al., 2019). While harm reduction strategies, like take-home naloxone kits, and sterile injecting equipment, and overdose prevention sites represent the standard-of-care available in the community around Nova Scotia, these were not sanctioned or reliably provided in Nova Scotia hospitals. The lack of hospital policy has contributed to the hospital harm described above, as no standards were established.

Here, we describe processes and outcomes of our community organizing work at the QEII Health Sciences Centre to improve substance use care, despite a lack of policy and institutional support. This includes “in-reach” of community-based services and the co-production of informal hospital substance use harm reduction policies and procedures by a multidisciplinary working group, including people with lived experience. We were able to improve inpatient hospital care by organizing ourselves and leveraging the resources that were already available in the community. This helps address an implementation gap, as these are evidence-based interventions in outpatient settings, which were not available for patients in hospital. Our work is now being used as a model across the province to promote consistent, compassionate hospital care for people who use drugs. This work has demonstrated that hospitals and health systems can learn from community-based harm reduction organizations who are already doing this work. Partnerships with people who use drugs and the organizations that support them are essential to the development of policies guiding care delivery.

## Harm reduction interventions and policy development

### Take-home naloxone kits in the emergency department and inpatient units

In 2017, the provincial Department of Health and Wellness established the Nova Scotia Take-Home Naloxone Program. This program provides free kits and training to respond to suspected opioid poisonings. Naloxone is now available at over 400 locations across Nova Scotia, including pharmacies, healthcare clinics, and community harm reduction organizations without a prescription or medical directive (Nova Scotia Take Home Naloxone Program, N.D.). However, the program did not include hospitals.

Initially, individual hospital-based physicians would obtain take-home naloxone kits from community pharmacies and distribute them to individual patients. While there were no policies facilitating take-home naloxone distribution from acute care, in early 2019 we simply registered the QEII hospital emergency department and internal medicine inpatient units as “distribution sites” for the provincial program, analogous to an outpatient health clinic or pharmacy. Distributing naloxone kits was optional among hospital staff, who were expected to complete online learning modules from the provincial program. Hospital pharmacists especially took initiative to get the training and ensure access. While implementation on the internal medicine unit happened quickly after manager approval, roll-out in the emergency department was delayed by several months due to management concerns about clinicians being expected to take on additional responsibilities outside their job description. One senior administrator also expressed concerns about “naloxone parties”, which reflected a moral panic at the time (Crabtree & Masuda, 2019).

### Distributing sterile drug preparation and injection equipment

While the QEII did not have any official policies or processes to support inpatient needle and syringe distribution, Mainline Needle Exchange had been doing informal hospital “in-reach” to support their clients for many years—though this was not generally known to hospital-based providers. Also available in Nova Scotia outpatient community pharmacies as of 2020 are “brown bags” of sterile injecting equipment, which are prepared by harm reduction organizations, like Mainline. The brown bags include 10 syringes, 10 alcohol swabs, a tourniquet, sterile water, and a few cookers to dissolve powdered and solid drugs for injection, as well as contact information for local harm reduction programs (MacLean, 2020).

**Fig. 1:**
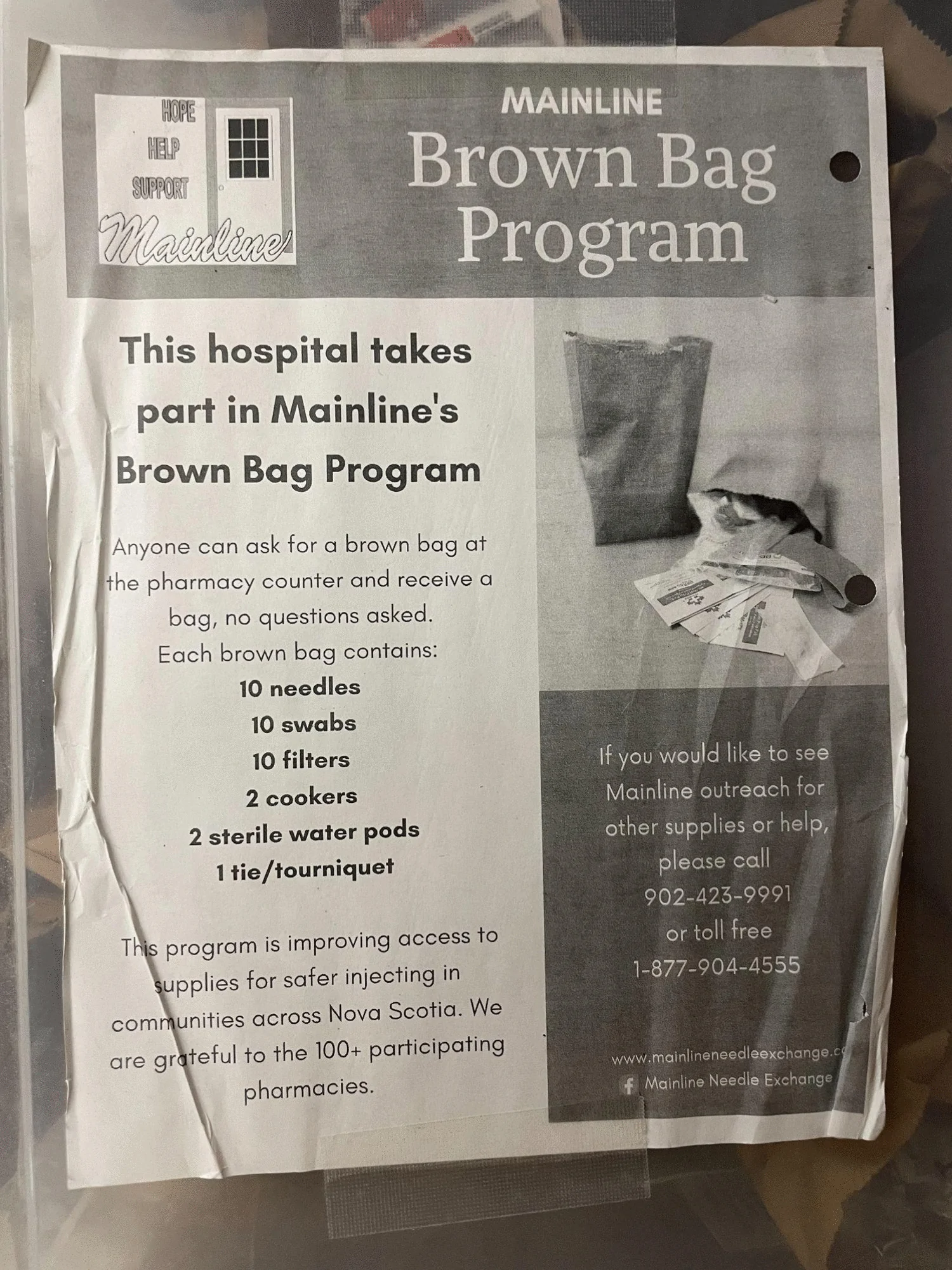
Sign describing Mainline Needle Exchange’s Brown Bag program, on top of a box containing sterile drug preparation and injecting equipment for distribution to patients at the Queen Elizabeth II Health Sciences Centre Emergency Department in Halifax, Nova Scotia

Like take-home naloxone kits, individual hospital-based physicians would obtain brown bags from community organizations and distribute them to individual patients. After brown bags became widely available in the community, an emergency physician began inviting Mainline to stock them in the QEII emergency department (Fig. 1). Clinicians began distributing these to ED patients being discharged home, and soon inpatient clinicians began retrieving them from the ED supply to distribute them to inpatients. However, without clear policy guidance, nurses and allied health providers were unable to routinely distribute equipment; awareness and access remained inconsistent. The new QEII Addiction Medicine Consult Service (see below) has been able to distribute equipment to their patients in hospital. For a brief time, senior leadership restricted this practice due to the lack of policy. This restriction was then reversed after complaints from consult service physicians.

### Withdrawal management and opioid agonist treatment

Until 2017, physicians working at the QEII would not routinely initiate opioid agonist treatment for opioid use disorder. In 2018, a group of hospital-based resident physicians began seeing inpatients after hours to support withdrawal and pain management and initiate opioid agonist treatment, while supervised remotely by community-based family physicians. This group soon became organized with weekly rounds and educational opportunities outside of traditional residency rotations. An evaluation of this unofficial addiction medicine consult service demonstrated that of 34 patients with untreated moderate-severe opioid use disorder, 82% initiated opioid agonist treatment in hospital and 89% of these patients continued after discharge (Brothers et al., 2021a). Adequately managing opioid withdrawal and pain allowed more patients with opioid use disorder to stay in hospital and receive needed medical care. We also developed and implemented a checklist to serve as an unofficial pre-printed order set to initiate buprenorphine in the emergency department and inpatient units, with a pathway for referrals and follow-up appointments at outpatient clinics (Dalhousie University Faculty of Medicine, n.d).

The informal service had issues with sustainability, as resident physicians graduated or left for exams or parental leave. Although the participating resident physicians had support from their program directors, they were threatened with disciplinary action from senior leadership for concerns about potentially practicing out-of-scope of their educational licenses. This led to further residents stepping away, and the informal service shutting down.

However, this experience and evaluation contributed to a successful business case and in 2023 an official hospital Addiction Medicine Consult Service began at the QEII, the first of its kind in Atlantic Canada. The service includes an interprofessional team of addiction medicine physicians, nursing, social work, peer support workers, and administrative support available for in-person consults at the QEII Halifax Infirmary campus and providing telephone support to healthcare providers elsewhere across the province.

### Injectable opioid agonist treatment (iOAT)

There were also inconsistencies between clinicians and inpatient units in approaching patients who required intravenous opioids (i.e. for whom oral opioids were insufficient) for treatment of pain or withdrawal. Some units restricted the intravenous use of liquid opioid formulations outside of critical care areas based on institutional culture (often referred to as a nonexistent “policy”), requiring patients on these units to receive oral opioid tablets, even if they continue to crush and inject them. This would lead to new bloodstream infections in hospital, and mistrust between patients and providers about “cheeking” and “diverting” medication.

In this context, some clinicians supported people who inject drugs to self-inject prescribed opioid or stimulant tablets while in-hospital (without pretending to swallow them), if they had capacity to decline to take the tablets orally as intended (Brothers et al., 2023b). There is no supervised consumption site in the QEII to ensure this can be done privately and hygienically, so patients would inject in their hospital rooms or bathrooms, or outside the hospital. However, some units enforced an existing policy that does not allow patients to self-administer controlled medications intravenously, so patients on those units had to put the tablets in their mouths. We sought guidance from the nursing regulatory college on our interpretation of the existing policies and policy exemption processes, and they said that they could not comment if there was no policy explicitly endorsing this practice of patient tablet self-administration. As a result, many nurses felt they could not safely apply these workarounds.

**Fig. 2:**
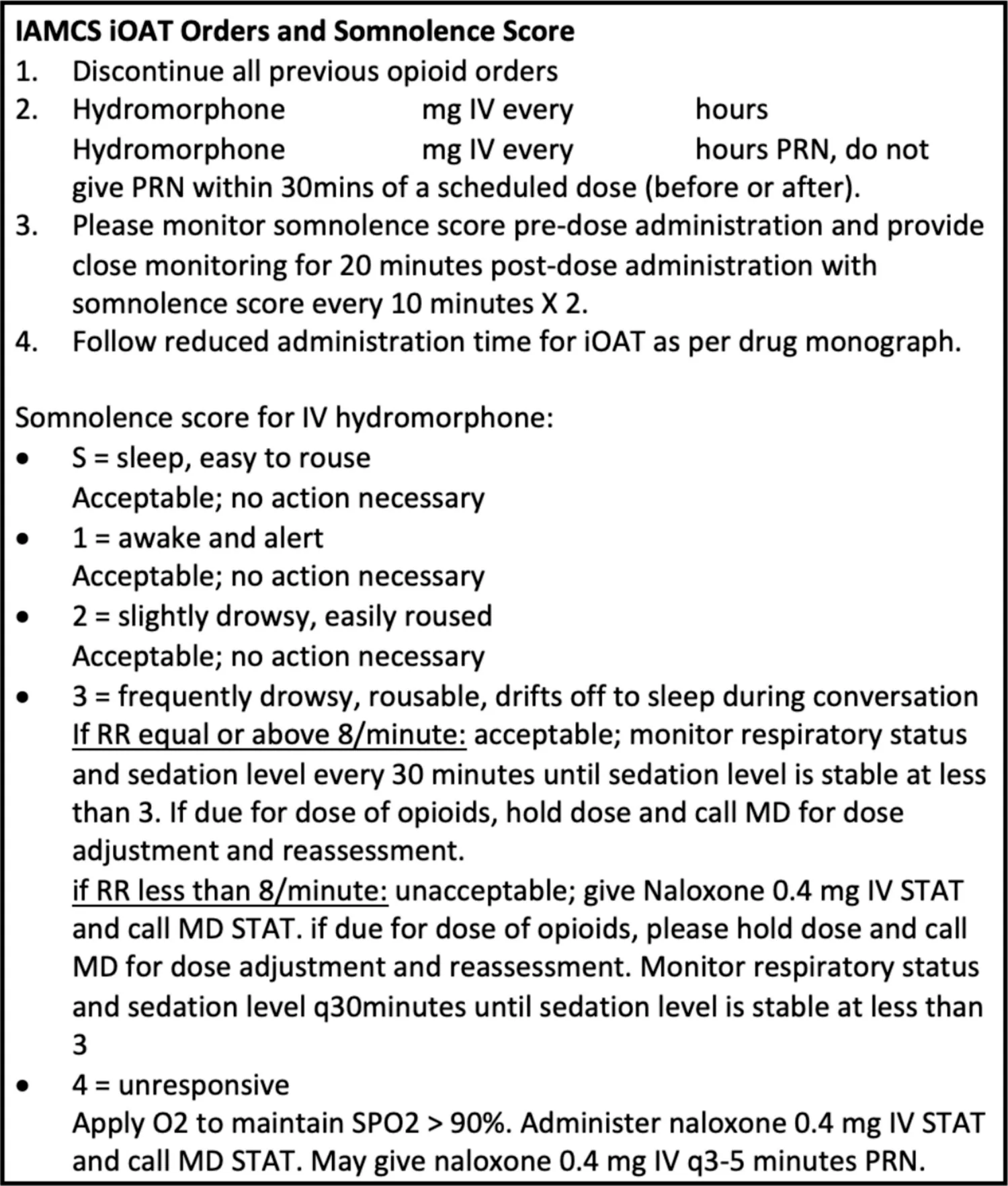
Injectable opioid agonist treatment (iOAT) orders with sedation scoring

The Addiction Medicine Consult Service and hospital pharmacy department worked together over several months in 2024 to have the institutional product monograph for liquid hydromorphone updated to include the Health Canada-approved indication for injectable opioid agonist treatment (Health Canada, 2019). This would be nurse-administered, in keeping with the existing institutional policies on administration of controlled substances. The group developed dosing guidance and a sedation monitoring approach, based on the Pasero Opioid-induced Sedation Scale (Pasero, 2009) (Fig. 2). This was incorporated into the monograph and approved by the Drugs and Therapeutics Committee. The Addiction Medicine Consult Service did education sessions with nurses while implementing this practice across the hospital, but so far there has been no province-wide rollout or change management—although the updated monograph is province-wide in scope.

In our clinical experience, patients were accepting of nurse-administered iOAT with intravenous hydromorphone when oral medications were insufficient. This is effective for pain control, provided more consistent dosing and monitoring which reduces risk of overdose, reduced the use of tablet medications, and avoided risks of tissue damage and infection from crushing and injecting tablets. It also practically eliminates any risk of medication sharing or selling (diversion). We can also facilitate antibiotics and investigations by timing them with scheduled iOAT doses. Process and outcome evaluations of this innovation are ongoing.

### Policy development on internal medicine inpatient unit

With growing harm reduction efforts and a changing practice culture, the need for institutional policies and procedures became more urgent. Conversations about harm reduction policy development for the internal medicine inpatient unit at the QEII began in January 2018. In the spring of 2018, the idea was presented to the unit’s quality committee. We also began education and training for hospital staff in 2018, inviting the community organization Mobile Outreach Street Health (MOSH) to present to medical residents, nursing, and allied health professionals. In January 2019, the internal medicine unit was officially registered as a distribution site with the take-home naloxone program. In January 2021, a multidisciplinary harm reduction working group was formed and conducted an environmental scan to understand what internal policies did exist to support or restrict certain practices (e.g. related to administering controlled medications) and what harm reduction policies existed elsewhere (e.g. Alberta Health Services, Providence Healthcare, Saint John Regional Hospital) (Brooks et al., 2024; Brothers et al., 2022; Chase et al., 2022; Lennox et al., 2021). In May 2021, this group began initial drafts of a philosophy of care document, standards of practice, and harm reduction policies for inpatients who use injection drugs.

Nursing leadership identified common challenging clinical situations on the unit, including scheduling (antibiotics administration, consultations, investigations); use and maintenance of peripherally inserted central catheters (PICCs); administration of controlled substances; sharps safety; and patient environment and belongings. The working group used these priority areas to frame guidance on standard operating procedures, acknowledging where policies did not exist and interpreting where they did. The working group drafted a model for policies to promote the use of evidence-based practices to instill a safer environment and reduce harms associated with substance use in hospital settings, while promoting dignity for patients through a non-judgmental approach. Aims were to promote harm reduction practices that are compassionate, patient-centred, and evidence-based, while promoting human rights, and limiting institutional liability by offering the community-based standard of care.

The working group has grown over the last few years, comprising representatives from nursing, social work, pharmacy, medicine, Mainline Needle Exchange, and people with lived experience. The group meets monthly to further develop the draft policies and discuss ongoing harm reduction efforts on the unit. It shares drafts with hospital- and community-based colleagues for further feedback and revision. Nursing leadership and health services manager members of the working group now regularly support leaders on other units in developing harm reduction-oriented care plans.

**Fig. 3:**
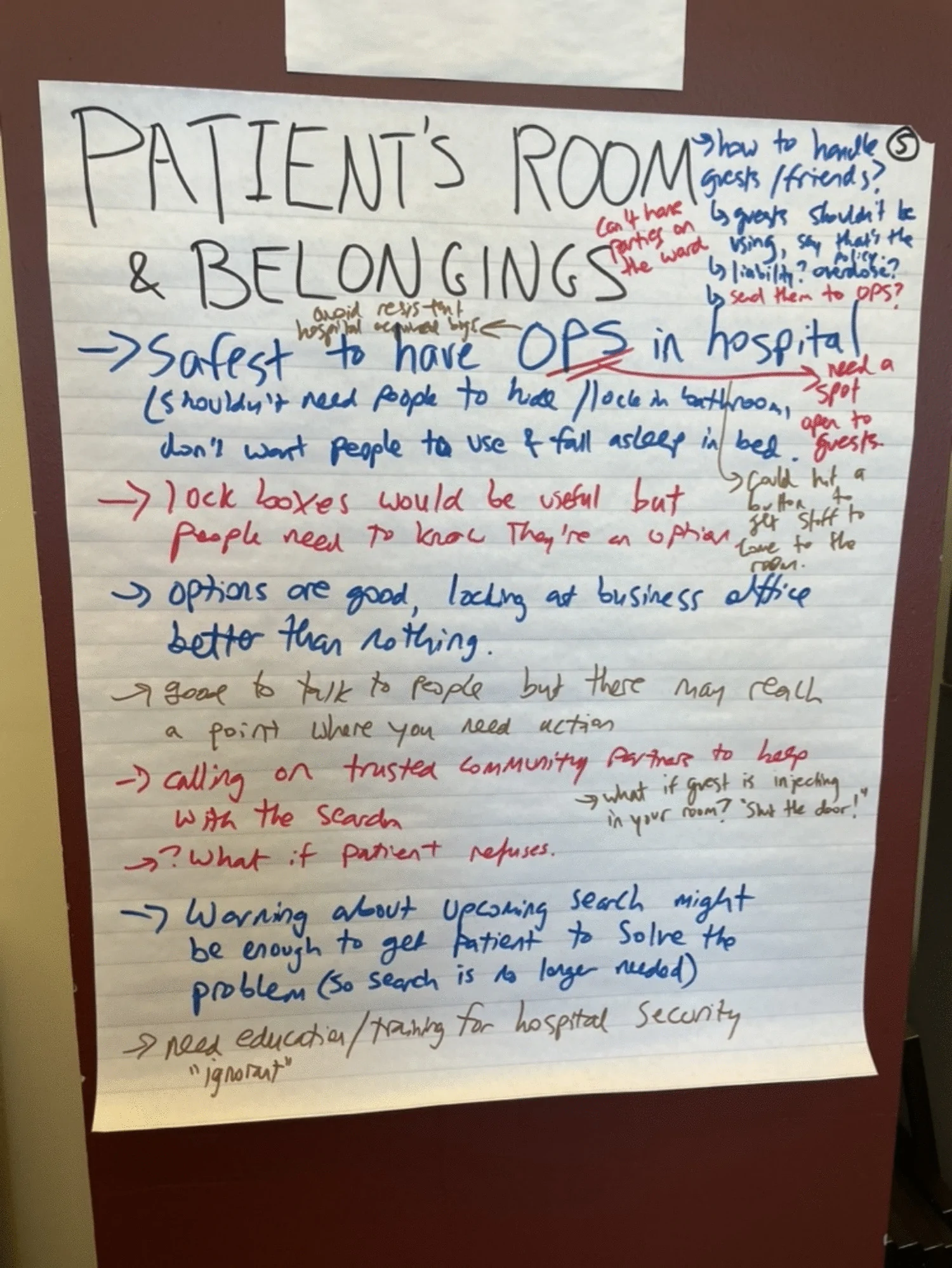
Flip chart notes reflecting discussions during community-based focused group at Mainline Needle Exchange

In December 2023, Mainline hosted a focus group where the proposed harm reduction policies were reviewed and discussed with six people who use drugs who had recently been hospitalized (Fig. 3). The draft policy was updated by the harm reduction working group to reflect the group discussion with patient partners with lived experience. Focus group participants felt our proposed approaches were feasible. Feedback from the focus group emphasized the importance of incorporating compassion and nonjudgment into policies and guidelines, especially around scheduling and time away from the ward. Additionally, the group highlighted the perceived benefits of harm reduction education, especially around safer use of PICC lines as well as the need for reliable access to safer injection equipment. Challenging existing policies around the administration of controlled substances, focus group participants advocated for their right to autonomy, self-determination, and safety, asking for the opportunity to self-administer medications (including by injection), and for dedicated spaces for supervised consumption of unregulated substances. They also requested a standardized approach to having visitors on the unit. The current version of the informal policy document is available as Supplement 1.

NSHA has now committed to establishing harm reduction policies across the organization, and establishing harm reduction practices as the standard of care, including take-home naloxone kits, needle and syringe distribution, and opioid agonist treatment (including iOAT). This work involves several members of our group. Our experience introducing community-based harm reduction services and co-designing harm reduction policies—drawing on input from multidisciplinary clinicians, community-based organizations, and people with lived experience—is directly informing the new provincial policies. This new harm reduction policy has been officially implemented on April 25, 2025. It endorses a harm reduction philosophy of care and explicitly supports as the standard-of-care distribution of sterile drug preparation and injecting equipment, take-home naloxone kits, access to opioid agonist treatment (including intravenous hydromorphone in hospital for opioid withdrawal), testing for sexually transmitted and blood-borne infections, and a Housing First approach to housing supports. The policy is publicly available at: https://policy.nshealth.ca/Site_Published/NSHA/document_render.aspx?documentRender.IdType=6&documentRender.GenericField=&documentRender.Id=109042.

## Discussion

Despite a lack of institutional policy or buy-in from senior leadership, harm reduction measures can be implemented in hospitals from the bottom up and from the outside in—by clinicians organizing, leveraging existing tools from community-based harm reduction organizations, and listening to people who use drugs. When the standard of care in the hospital does not meet the standard of care in the community, this becomes an issue of equity and patient safety. Health systems have a duty to provide high-quality care and to accommodate people with substance use disorders, and patients have a right to high-quality healthcare. Simply by providing the standard-of-care available in the community, we can help people stay in hospital to access often life-saving medical care, reduce their drug use or use drugs in less dangerous ways, and decrease their risk of infections and overdose while hospitalized (Crowther et al., 2023; Fraimow-Wong, 2024).

While harm reduction measures can be effective, institutional responses are still needed for standardization and sustainability, especially as certain barriers to providing harm reduction-oriented care cannot be overcome without support from institutional policy. Without policies, care provision lacks consistency between units and even between providers on the same unit. For example, there have been differences in the distribution of naloxone kits and sterile injecting equipment between units because these were not mandated at an institutional level with standard procedures. We feel substance use care should be incorporated into hospital accreditation standards in Canada (Englander & Davis, 2022). The provincial nursing regulatory college told us they could not support nurses distributing sterile injecting equipment in hospital unless there is an explicit institutional policy to support this (even though there were no policies restricting it). Our group organized to learn from community-based organizations (including Mainline, MOSH, and Direction 180) and train ourselves and each other; however, sustained and ongoing capacity development requires an institutional approach and dedicated resources. Throughout this process, we engaged with the emerging evidence for hospital-based substance use care interventions, citing empirical studies in presentations to leadership, education sessions, informal policy/procedure documents, and business case drafts (CRISM 2020; Brothers et al., 2021a; Crowther et al., 2023; Dong et al., 2020; Lennox et al., 2021). We also learned from other hospitals with institutional commitments and official policies supporting harm reduction, including Saint John Regional Hospital (New Brunswick) (Brothers et al., 2022), Royal Alexandra Hospital (Edmonton, Alberta) (Brooks et al., 2024; Dong et al., 2020; Kosteniuk, 2021), St. Paul’s Hospital (Vancouver, British Columbia) (Chase et al., 2022; Nolan et al., 2022), and Casey House (Toronto, Ontario) (Miskovic et al., 2018).

Our group is now focused on sustaining these initiatives, expanding to other hospital sites in Nova Scotia, and consulting with the provincial health authority on the development and implementation of the newly implemented province-wide harm reduction policies for healthcare settings, and co-designing further improvements—for example, an approach to hospital-based supervised consumption services. This includes a push for permanent funding to sustain interventions and support for people who use drugs to have leadership roles in care, policy design, and care delivery. We are also undertaking a participatory qualitative study, exploring our group’s experiences trying to deliver harm reduction-oriented care without institutional support.

Our approach complements others who have recently described initiatives leveraging community resources and partnerships to implement harm reduction interventions and policies in hospital (Goff et al., 2024; Lokko & Avor, 2024; Perera et al., 2022). For example, Hyde and colleagues (2023) describe a phased approach to hospital-based harm reduction initiatives in a tertiary care hospital in Winnipeg, Manitoba, comprising education sessions, development and institutional approval of a harm reduction philosophy-of-care statement, and distribution of sterile drug preparation and injecting equipment to hospital inpatients. Their working group included community harm reduction organizations, from which they obtained drug preparation and injecting equipment to distribute to hospital patients and invited experts to deliver education sessions. As part of a community-based research project, Wickremsinhe and colleagues (2024) developed hospital guidelines to improve management of opioid withdrawal and access to opioid agonist treatment in hospitals in England. Their approach also involved a multidisciplinary working group, local champions, and leveraging existing resources and relationships with organizations outside the hospital setting including a local community advocacy organization.

Implementation of harm reduction interventions in hospital and development of informal harm reduction policies to guide care have been novel in Nova Scotia and serve as a significant step toward providing more effective and compassionate care to patients who use drugs in hospital. This project serves as an example of how people can come together under a shared vision and collaborate to make impactful changes. Communities at other hospitals and health systems could follow this project’s approach, adapted to their local context.

## Implications for policy and practice

### What are the innovations in this policy or program?

- We developed coalitions of hospital-based healthcare providers and community harm reduction organized to improve hospital substance use care without formal policy and/or institutional support.
- This included updates to drug monographs (e.g. hydromorphone injectable opioid agonist treatment; iOAT), informal addiction medicine consult services, take-home naloxone kits available in hospital, and distribution of sterile injecting equipment in hospital.
- We pushed the provincial health authority to develop and implement province-wide harm reduction policies for healthcare settings, informed by our work.

### What are the burning research questions for this innovation?

- To evaluate harm reduction interventions, we would want to explore the impact of these interventions on patient outcomes, particularly their ability to stay in hospital and receive treatment, to not leave against medical advice, as well as patient satisfaction with treatment.
- It would also be helpful to explore staff perspectives, and barriers and facilitators to the implementation of harm reduction interventions, like education, comfort in providing harm reduction interventions, and the effect of lack of institutional/policy support on ability to provide harm reduction interventions.

## Supplementary Information

Below is the link to the electronic supplementary material.

ESM1 (DOCX.4.84 MB)

## Data Availability

Not applicable.
