# Supplementary material for: Improving hospital substance use care from the ground up and from the outside in
Source: Can J Public Health. 2026 Mar 20;117(5):921–30. doi: 10.17269/s41997-026-01154-5 (PMC13680350; doi:10.17269/s41997-026-01154-5)
Supplement: Supplementary file 1 [file 41997_2026_1154_MOESM1_ESM.docx]

**Optimizing Inpatient Care for Patients with Substance Use Disorders:**

**A Harm Reduction-Focused Guidance Document**

Unit 8.2, Medical Teaching Unit (MTU)

Halifax Infirmary QEII, Nova Scotia Health Authority

For the purposes of this document, the term “substance use” refers to use of illicit drugs, alcohol, and prescribed medications in a non-prescribed manner. “Substance use disorder” is a chronic (and often relapsing) health condition, characterized by substance use in a pattern associated with significant impairment or distress.

For the purposes of this document, the term “health care team” refers to all individuals involved in the care of the individuals with substance use disorders during their admission, including, but not limited to:

- Physicians (including consulting physicians, and all residents and fellows)
- Nurses
- Social workers
- Physiotherapists
- Occupational therapists
- Pharmacists
- Dieticians
- Respiratory therapists
- Continuing care staff
- Spiritual care providers
- Care Team Assistants
- All learners (including, for example, medical, nursing and physiotherapy students)
- Administrative and support staff on the ward.

1.0 **Introduction**

**1.1 Purpose**

The purpose of this guidance document is to provide direction and clarity to health care team members around their roles and responsibilities when providing care to patients with substance use disorders during their admission to Unit 8.2. This guidance document is focused on promoting an evidence-based, harm reduction-oriented approach to care. It is aimed at:

- Promoting dignity and respect for all patients
- Recognizing that patients may have dependencies on alcohol or other substances
- Promoting a non-judgmental approach to caring for individuals with substance use disorders
- Promoting a safe environment for patients, families and staff
- Reducing the harm associated with alcohol/substance use

**1.2 Statement**

Unit 8.2 has an ethical and legal duty to provide care to all patients and to ensure the safety of patients, families, visitors, staff, physicians and volunteers. This document aims to promote safety and to reduce harm while recognizing it is not always possible to control or eliminate the use of all alcohol or substances on hospital premises when individuals are struggling with substance use disorders.

2.0 **Scope**

This guidance document applies to all Unit 8.2 staff.

3.0 **Overview**
1. *Assessment*. The patient’s health care team will assess the patient’s knowledge about the risks of substance use and stage of behavior change (pre-contemplative, contemplative, action, maintenance) in relation to their substance use. The health care team should also explore the patient’s goals related to substance use (ie. reducing use, reducing harms of use, abstinence). Their stage of change and related goals should be documented in the chart and the care that follows during their admission should align with their identified goals.
2. *Autonomy*. Patient autonomy should be upheld by ensuring individuals with substance use disorders are heard and respected in decision-making around their care, even when their decisions or actions do not align with the recommendations of care providers. Each patient should be provided with care that respects their dignity, humanity and autonomy. As care providers, we need to meet individuals where they are at during their stay.
3. *Care plans*. Where appropriate, individualized care plans will be developed by the health care team with the following guidelines:
a. Care planning is a collaborative team effort that includes the patient, inpatient Health Care Team, (including the Addiction Medicine Consult Service when appropriate) community-based care providers and family/support people involved in the patient’s life, as identified by the patient.
b. Care planning may be done through team conferences and should include all team members directly involved in the patient’s care, when available.
c. While there may be times when the care team needs to discuss care without the patient’s participation, the patient should be included in decision-making related to their care whenever possible.
d. Follow up plans and continuity of care are to be arranged and documented as part of the care plan.
4.0 **Guidance for Frequently Encountered Clinical Scenarios**
I. Scheduling and Communication of Treatments/Investigations/Consultations
II. PICC Line Maintenance
III. Administration of Controlled Substances
IV. Sharps Safety
V. Off- Ward Time and Discharge Planning
VI. Patient Environment
    4.1 **Scheduling and Communication of Treatment/Investigations/Consultations**

**Goal:** To obtain optimal medical treatment by ensuring that patients are available for planned treatments, investigations and assessments with consulting teams.

**Intervention:**

1. Planning & Communication:
   a. The assigned nurse will talk to the patient about the plan for the day (e.g. antibiotic administration, investigations, consultations) at each shift
   b. When possible, inpatient staff will ensure there is a documented plan for how to contact the patient if not in room (e.g., cell phone number, consent to communicate with community supports)
   c. Investigations: Whenever possible, the inpatient unit will communicate with booking offices at the start of the day and inform the patient of the approximate timing and importance of being available.
   d. Consulting Services: If a consulting service is having difficulty meeting with the patient, they can be invited to arrange a time to meet with the patient.
   e. Antibiotic Administration:
      i. The patient should be notified if a dose is to be delayed due to staffing reasons. If the dose is to be delayed due to staffing reasons, this should be communicated to the patient.
      ii. If the patient does not return to the unit for a dose and more than 2 hours have elapsed since the medication was scheduled to be administered, and this is the first time this has happened, the nurse should attempt to contact the patient. The nurse should notify the medical team if they are unable to contact the patient and a dose has been missed.
      iii. When the patient returns to the unit following a missed antibiotic dose, they should be welcomed back on the unit with unconditional positive regard and the medical team should be notified. The medical team can then provide direction about when the next scheduled antibiotic dose should occur (now or held until the next scheduled dose).
      iv. If there is a pattern of missed doses (e.g.+2 or more doses given >2 hours after scheduled time due to unexpected patient absence):
          1. Members of the care team should meet with the patient to discuss the missed doses, with the goal of understanding root causes and finding solutions (ie. patient is having ongoing unaddressed cravings and associated substance use that is taking them off the ward, social stressors, feeling stigmatized/unwelcomed on the unit, etc.).
          2. Revising the care plan (e.g. a new approach to withdrawal management) and/or care agreement can be explored to clarify the individual’s health care goals for the admission and identify how the care team can best support the individual in achieving these goals.
   4.2 **PICC Line Care and Maintenance**

**Goal:** Minimize use of PICC lines for non-prescribed substance administration when possible, exploring alternatives with the patient (such as consuming substances through non-injecting routes) and/or weighing the risks and benefits of having a PICC line versus transitioning to an alternative medication (e.g., oral antibiotics instead of IV antibiotics if this is the purpose of the PICC). Should the patient use the PICC line for injecting non-prescribed substances, education and teaching will be provided to ensure proper care and safe use of the lines.

Note that some substances need to be heated with an open flame to dissolve optimally, but open flames (i.e., lighters) cannot be used in hospitals.

**Interventions**:

1. Provide alcohol swabs, sterile flushes, fresh caps, and dressings. Provide masks and gloves and encourage use while touching PICC and/or changing caps.
2. Offer education and support, answering questions the patient may have regarding line care and safety.
3. Offer the patient a naloxone kit and education around its use. Patients who report only stimulant use should still be offered a naloxone kit.
4. If the PICC line is occluded, contact the physician team to inform them that the patient is without IV access; consider means of treatment and/or attempt peripheral access.
   4.3 **Administration of Controlled Substances**

“Controlled substances” or “controlled medications” are defined as per Canada’s Controlled Drugs and Substances Act (CDSA), as medications that fall under the categories of:

- Narcotics (including opioid analgesics and opioid agonist treatment medications such as methadone or buprenorphine [“Suboxone”])
- Benzodiazepines
- Amphetamines (including methylphenidate, dextroamphetamine, and methamphetamine)

**Goal:** To safely administer controlled substances following assessment, evaluation and documentation using a harm reduction-focused philosophy.

**Interventions:**

1. When a patient is identified as already being on, or newly prescribed, a controlled substance in the context of a substance use disorder, the health care team is encouraged to involve the Addiction Medicine Consult Service early in the admission. The early involvement of the Addiction Medicine Consult Service will facilitate the following:
   a. Continuity of care in management of controlled substances
   b. Early identification of transitions, when appropriate and if the patient is interested, to opioid agonist treatments (e.g., methadone, buprenorphine, slow-release oral morphine)
   c. Transition from hospital to community-based care providers around the time of discharge, including communication with the patient’s usual care provider when appropriate.
2. For most patients with substance use disorders, the care plan will involve controlled medications administered by the nurse.
3. If patients continue to inject substances despite involvement of the Addiction Medicine Consult Service, in exceptional circumstances (and if deemed necessary by the medical team to support patients to stay in hospital and receive necessary medical care), orders will be written that the bedside nurse is not required to witness a patient taking a controlled medication by mouth.
   a. When this is made a part of an individual patient’s care plan, it is recommended that the Addiction Medicine Consult Service be consulted.
   b. This care plan should be routinely re-evaluated, with transitions to oral and/or nurse-administered medications when possible.
   c. Note that this guidance establishes exceptions to the existing Nova Scotia Health policy, “Self-Administration of Medication (SAM)” (MM-MA-020), which states, “Narcotics and benzodiazepines are not to be left in a patients’ room for self-medication purposes.”
4. *Assessment*: prior to administration of a controlled medication, the bedside nurse will assess the patient’s level of pain, level of consciousness, and respiratory rate/saturations, as appropriate.

- At the discretion of the medical team, orders can be written to hold certain controlled medications if clear parameters are met (ie. if patient has a respiratory rate <8, if patient is difficult to rouse). When these parameters are met, the medical team should be notified and the patient should be assessed as deemed appropriate.

5. *Education*: the care team should provide education regarding purpose of controlled medication, intended route, potential potency difference (due to either concentration or route of administration), and expected effects, and inform patient that nursing staff will complete a post-administration evaluation 30-60 minutes following dose.
6. *Evaluation:* Nursing staff will return to assess patient’s response to medication 30-60 minutes post- administration. This will include assessment of pain, level of consciousness and respiratory rate/oxygen saturations as deemed appropriate for the administered controlled substance and its indication.
7. If unable to see the patient (I.e. patient is in the bathroom), evaluation may be completed initially via communication through the door. If the patient is able to carry a conversation, their airway and level of consciousness would be considered adequate until further complete assessment within 5 minutes.
8. *Documentation*: Nursing team will document administration of the medication in the progress notes, as well as in the patient’s medication administration record (MAR). Documentation should include: pre-administration assessment education provided, patient’s understanding, patient’s response to administration and post-administration evaluation.
9. All efforts should be made to ensure timely administration of controlled medications to avoid withdrawal and other adverse effects. This includes considerations such as:
   a. Ensuring medications are ordered for the morning if the patient is admitted to hospital overnight
   b. Obtaining accurate history of use (including verbal history) to ensure appropriate amounts prescribed. For verbal history of non-prescribed substances, healthcare providers should use the same logic as other medications (i.e. if they don’t know dose of heart medication start low and give more if needed, should be same for controlled substances).
   4.4 **Sharps Safety**

**Goal:** Safe disposal and storage of sharps to ensure a safe care environment for patients and workplace for staff, while facilitating access to sterile injecting equipment for patients to reduce risks of infection.

**Interventions:**

1. Sharps containers will be provided and maintained in patients’ rooms.
2. Unit managers should ensure new staff are aware of availability of harm reduction supplies and sharps containers.
3. To ensure safety of others, a unit aide should be notified when sharps containers are full. This can be done by the patient or any members of the care team.
4. Staff should always perform a point of care risk assessment for potential workplace hazards, including risk of needlestick injuries, and proceed accordingly.
5. Encourage the patient to keep all supplies in a locked storage bin provided and reinforce the importance of sharps safety as needed. Do not remove patient’s sterile drug preparation and injecting equipment, as this can lead to use and sharing of contaminated equipment and increase risks of infection.
6. Sterile drug preparation and injecting supplies (i.e., insulin syringes, filters, cookers, tourniquets, and sterile water) are available for all inpatients and should be offered and provided when patients have known substance use. Communication with the patient is key to ensure that appropriate and adequate supply can be distributed based on patient familiarity and preference. They can be found in brown bags in the 8.2 medication room and in Pod 5 of the Halifax Infirmary Emergency Department – they are provided by Mainline. Any member of the health care team can pick up clean supplies and provide them to MTU patients. Once patients are admitted, they can access these supplies on the unit. For individual specific or additional supplies and for outreach support, Mainline can be contacted by the patient or in the inpatient care team (with patient consent).
7. Patients using any unregulated substances (including opioids, stimulants, and benzodiazepines) should be provided with a naloxone kit and education around its use. Naloxone kits can be found in the 8.2 medication room. If none are available there, further kits can be found in Pod 5 of the Halifax Infirmary Emergency Department.
   4.5 **Off-Ward Time**

**Goal:** To optimally engage patients in ward-based care during their admission, minimizing off-ward time that negatively impacts optimal timing of medication administration and reduces encounters with the medical team (I.e. off-ward time leading to missed physician assessments on daily rounds).

Note that it should not be usual or common practice to contact police for a “wellness check”, as many patients who use drugs are criminalized for their substance use and have experiences of traumatic encounters with police.

**Interventions:**

1. Provide the patient with a timeline of next medication doses and any expected upcoming tests at the beginning of each shift. Some patients may need several reminders and additional strategies including written notes.
2. Prior to the patient leaving the unit, review contact information for that day as it may change depending on where they are going, and who they are with. Record that contact number in the progress notes.
3. If the patient does not return at the intended time, attempt to contact them and determine if a support person should also be called. If unable to contact the patient, the medical team should be notified (with the goal of ensuring the safety of the patient) and Code Yellow should be called.
4. If/when the patient returns:
   a. The patient should be welcomed back to the unit.
   b. Members of the care team should meet with the patient to explore the reasons for their prolonged absences, with the goal of understanding root causes and finding solutions (I.e. patient is having ongoing unaddressed cravings and associated substance use that is taking them off the ward, social stressors, feeling stigmatized/unwelcomed on the unit, etc.)
   c. With repeated absences, a care plan and/or care contract can be explored to clarify the individual’s health care goals for the admission and identify how the care team can best support the individual in achieving these goals.
   d. If patient comes back from being off ward and has used a substance, this may result in a delay in regularly scheduled dosing of controlled substances if there is a concern of potential toxicity
5. If the patient does not return to the unit after 24 hours, attempts should again be made by the care team to contact the patient and identified support person, if one is known.
6. If the patient cannot be reached and does not return, they will be discharged, and community care providers will be notified.
7. If individual is known to Mobile Outreach Street Health (MOSH) call:
   a. MOSH Nursing Line - **902-802-3332** (available daily between 9am-7pm) and/or
   b. MOSH Street navigator – **902-209-6517**
8. To gather information about community whereabouts, the care team can also call Mainline – **902-423-9991**
9. Unplanned Discharge: If the patient does not return to the unit after 24 hours, attempts should be made by the care team to contact the patient. A Code Yellow should only be initiated if the criteria for this procedure are met (lacking capacity, risk identified). If the patient cannot be reached and does not return, they will be discharged, and community care providers (e.g. Mobile Outreach Street Health [MOSH]) will be notified with an updated outpatient management plan (e.g., oral antibiotics available at a local pharmacy and potential follow-up appointments). Note that it is not typical or expected to contact police to perform a “wellness check” in most circumstances, and follow-up can be much more effective through community organizations (e.g., MOSH).
   4.6 **Patient Environment:**

**Goal:** To provide patient autonomy in patient’s bed-space area. Nursing will only go into a patient’s bed-space and tidy up on request of patient or in the event of an identified safety concern.

**Interventions:**

1. Education provided to patient to secure any personal belongings using storage bins provided or encourage patient to send items home (if possible, recognizing that not all patients will have a safe space to store personal belongings, or person to take it for them). Patients may store valuables at the hospital business office while they are admitted, but these can only be withdrawn during business hours.
2. If an unknown substance is visible in the patient’s bed-space area, this could be an opportunity to assess patients’ substance use and withdrawal needs.
3. Patients should be asked to keep personal substances away, where it would be not be accidentally accessible by other patients. Patients can be offered a locked box with key.
4. In most circumstances, the presence of illegal/unregulated substances alone does not constitute a safety concern and would not automatically lead to a search of patients’ belongings nor automatically need reporting to security.
5. If there is a safety concern related to illegal/unregulated substances (e.g., substances found out in the open without an owner; patients unable to keep substances away and secured; open flame), healthcare providers should take a non-judgmental approach and discuss alternative options that do not pose a safety concern with the patient. If the safety risk continues, it should be reported to the physician team to discuss with patient.
6. If the safety risk is ongoing after physician intervention, health care providers may need to alert security as per policy Search of Patient Care Areas, Individual and/or Personal Property (CH20-027) (link available in 5.0 Related Documents section below) to dispose of substances. This policy clarified in section 2.1.9 that whenever possible the search of a patient’s belongings should be “in the presence of the patient and in the most private location available in the circumstances”.
7. If there are needles are found in the patient’s bed-space area, the patient – if present – should be asked to dispose of open/used needles safely using the sharps container provided and to put away unused needles so it is not out in the open. If the patient is not present, used or open needles should be disposed of and the patient should be reminded of the importance of safely storing and disposing needles to reduce potential harms to patients and staff. Sterile (i.e., unopened) needles could be put away in patient’s bedside table until they return.
8. Patients with substance use disorders may have visitors, (chosen) family and friends, who also have substance use disorders. We support patients having visitors in the hospital, consistent with NSHealth’s policy on family presence and essential care partners. While visitors are not under our care, we nonetheless recommend approaching visitors with the same principles as engaging with patients (above).
5.0 **Related Documents:**

- Philosophy of Care for Patients with Substance Use Disorders
- Search of Patient Care Areas CH20-027 available at: [*http://policy.nshealth.ca/Site_Published/DHA9/document_render.aspx?documentRender.IdType=6&documentRender.GenericField=&documentRender.Id=49554*](http://policy.nshealth.ca/Site_Published/DHA9/document_render.aspx?documentRender.IdType=6&documentRender.GenericField=&documentRender.Id=49554)
- Self-Administration of Medication (SAM) MM-MA-202 available at: <https://policy.nshealth.ca/Site_Published/NSHA/document_render.aspx?documentRender.IdType=6&documentRender.GenericField=&documentRender.Id=69301>
- Family presence AD-QR-020 available at: <https://policy.nshealth.ca/Site_Published/Provincial/document_render.aspx?documentRender.IdType=6&documentRender.GenericField&documentRender.Id=69516>
- Interacting with Law Enforcement AD-AO-095 available at: https://policy.nshealth.ca/site_published/nsha/document_render.aspx?documentRender.IdType=6&documentRender.GenericField=&documentRender.Id=108959

**REFERENCES/RESOURCES**

1. Allen D, Brouwer J, Dong K, et al. Management of substance use in acute care settings in Alberta: guidance document. Edmonton: Canadian Research Initiative on Substance Misuse (CRISM); 2020. Available at: <https://crismprairies.ca/management-of-substance-use-in-acute-care-settings-in-alberta-guidance-document/>
2. Brothers TD, Fraser J, Webster D. Caring for people who inject drugs when they are admitted to hospital. CMAJ. 2021 Mar 22;193(12):E423-E424. doi: 10.1503/cmaj.202124. PMID: 33753366; PMCID: PMC8096383. https://doi.org/10.1503/cmaj.202124
3. Rachlis, B.S., Kerr, T., Montaner, J.S.G., Wood, E. (2009). Harm reduction in hospitals: is it time? Harm Reduction Journal. 2009. 6:19.
4. McCall J, Pauly B. Sowing a seed of safety: providing culturally safe care in acute care settings for people who use drugs*. J Mental Health Addic Nurs* 2019*;*3*:*e1*–*7
5. A Harm Reduction Approach, Alberta Health Services, August 2019. Available at: <https://www.albertahealthservices.ca/assets/info/hrs/if-hrs-a-harm-reduction-approach.pdf>
